# The Suppressive Effect of Leucine-Rich Glioma Inactivated 3 (LGI3) Peptide on Impaired Skin Barrier Function in a Murine Model Atopic Dermatitis

**DOI:** 10.3390/pharmaceutics12080750

**Published:** 2020-08-10

**Authors:** Ui Seok Kim, Jin Woo Park, Eon Sub Park, Joon Seok Bang, Tae Woo Jung, Dong-Seok Kim, A. M. Abd El-Aty, Jong Hyuk Lee, Ji Hoon Jeong

**Affiliations:** 1 Department of Pharmacology, College of Medicine, Chung-Ang University, Seoul 06974, Korea; kuskus92@naver.com (U.S.K.); cparkjinwoo@naver.com (J.W.P.); twjung@cau.ac.kr (T.W.J.); 2 Department of Pathology, College of Medicine, Chung-Ang University, Seoul 06974, Korea; esp@cau.ac.kr; 3 College of Pharmacy, Sookmyung Women’s University, Seoul 04310, Korea; jsbang@sookmyung.ac.kr; 4 Department of Biochemistry, College of Medicine, Chung-Ang University, Seoul 06974, Korea; ds_kim@cau.ac.kr; 5 Department of Pharmacology, Faculty of Veterinary Medicine, Cairo University, Giza 12211, Egypt; abdelaty@staff.cu.edu.eg; 6 Department of Pharmaceutical Engineering, College of Life and Health Science, Hoseo University, Asan 31499, Korea

**Keywords:** atopic dermatitis, leucine-rich glioma inactivated 3 (LGI3) peptide, keratosis, skin barrier, filaggrin

## Abstract

This study aimed to restore the skin barrier function from atopic dermatitis (AD) via treatment with leucine-rich glioma inactivated 3 (LGI3) peptide. Male NC/Nga mice (7 weeks, 20 g) were randomly allocated into three groups (control, AD, and LGI3 group). After induction of AD skin lesions with *Dermatophagoides farinae* ointment, mice were treated with LGI3. The clinical score of AD was the highest and the dorsal skin thickness was the thickest in the AD group. In contrast, LGI3 treatment improved the clinical score and the dorsal skin thickness compared to the AD model. LGI3 treatment suppressed histopathological thickness of the epithelial cell layer of the dorsal skin. LGI3 treatment could indirectly reduce mast cell infiltration through restoring the barrier function of the skin. Additionally, the filaggrin expression was increased in immunohistochemical evaluation. In conclusion, the ameliorating effect and maintaining skin barrier homeostasis in the AD murine model treated with LGI3 could be attributed to complete re-epithelialization of keratinocytes. Hence, LGI3 might be considered as a new potential therapeutic target for restoring skin barrier function in AD.

## 1. Introduction

Atopic dermatitis (AD) is the most common chronic inflammatory skin disease, which affects at least 15% of children. The disease is caused by a complex interaction between genetic, skin, immune, and environmental factors [1,2,3,4,5]. AD skin appears frequently dry and shows severe itching when coming into direct contact with an irritant compared to healthy skin. The disease is classified into two categories based on clinical features: acute and chronic AD. In acute AD, the symptoms are severe pruritus, erythematous papules associated with excoriation and severe exudative lesions. On the other hand, chronic AD undergoes tissue remodeling due to chronic inflammation. Chronic AD skin lesions are associated with thickened plaques with wrinkled lichenification, increased collagen deposition in the dermis, and dry fibrotic papules [5].

Although the exact factors implicated in the pathogenesis and pathophysiology of AD (causing the initial inflammatory response) has not yet been well characterized, multiple studies have shown that skin barrier abnormalities and related immune responses are the principal mechanisms proposed to explain the pathogenesis [5,6,7,8,9]. Healthy skin barrier is the first line of defense against various allergens including pollen, irritant, and microorganisms (such as house dust mites). However, patients with AD are more susceptible to the development of contact sensitization to minor allergens [10,11,12]. Skin barrier dysfunction caused by both genetic variation and acquired impairment plays a central role in the initiation of AD. For instance, filaggrin (filament aggregating protein, FLG), a protein associated with keratin filaments in epithelial cells, has emerged as the most likely genetic risk factor involved in AD [10,13,14,15,16,17]. Thus, the FLG gene mutation (loss of function mutation or frame shift mutation) may directly damage the skin barrier and immunoglobulin E (Ig E) sensitization through the damaged skin barrier [18,19]. Further to that, transepidermal water loss results in the skin being dry, itchy, thickened, and wrinkled [9]. Therefore, proper treatment is necessitated to ameliorate the AD skin lesions and restore the skin barrier function. Leucine-rich repeat LGI family member 3 (LGI3) is exclusively expressed in various skin cells as well as brain and adipose tissues [20]. Lee et al., have reported that upregulation of LGI3 expression were observed in ultraviolet B-stimulated human keratinocytes [21]. LGI3 promotes keratinocyte differentiation, which is an important step in skin barrier establishment and maintenance [22,23]. Treatment with LGI3 peptide would promote the expression of FLG in a concentration-dependent manner including other proteins such as involucrin, loricrin, and transglutamainase-1, which are important components for skin barrier [22]. These results suggest that the LGI3 peptide may facilitate AD skin healing processes through enhancing keratinocyte differentiation, which maintain the skin barrier homeostasis. Therefore, LGI3 may be a potential therapeutic strategy for treating skin barrier disruption.

To the best of the authors’ knowledge, there are no specific reports regarding LGI3 alleviating AD skin lesions. Therefore, the aim of this study was to evaluate and investigate the recovery of skin barrier in AD by using the LGI-3 peptide as a therapeutic agent.

## 2. Materials and Methods 

### 2.1. Animals

Seven week old NC/Nga mice (male, 15~20 g) were procured from the Central Laboratory Animal (Seoul, Korea). The experimental mice were housed in a pathogen-free cleanroom of class 10,000, with a room temperature of 22 ± 2 °C, humidity of 55 ± 5%, and 12 h–12 h light/dark cycle with free access to food (Purina, Korea) and water. Animals were adapted (to this standard laboratory conditions) for a week before initiating the experimental work. Experiment 1: All animals were randomly assigned into two groups (n = 6 for each group): Control group: not suffer from AD and received no treatment; AD group: suffered from AD and received treatment with mixture of 99% phosphate-buffered saline (PBS) (Sigma, St. Louis, MO, USA) and 1% polyethylene glycol 400 (PEG 400, Sigma). Experiment 2: All animals were randomly assigned into three groups (n = 7 for each group): Control group: not suffer from AD and received no treatment; AD group: suffered from AD and received treatment with mixture of 99% PBS (Sigma) and 1% PEG 400 (Sigma); and the LGI3 group: suffered from AD and received treatment with 1 mg/mL LGI3 peptide (Peptron, Daejeon, Korea) and a mixture of PBS and PEG 400. All experimental procedures were performed following the Institutional Animal Care and Use Committee of Chung-Ang University (Approval number: 2019-00026, 7 May 2019). Upon completion of the study period, all experimental mice were sacrificed under inhalation anesthesia with diethyl ether after an overnight (12-h) fast.

### 2.2. Induction of Atopic Dermatitis

Biostir AD ointment (solid form) was acquired from Central Laboratory Animal (Seoul, Korea). The ointment’s ingredient is *Dermatophagoides farinae* (*D. farinae*), which is the best characterized house dust mite allergens. The allergens in this ointment cause severe pruritus, the main symptom of atopic dermatitis [24]. Ointment was kept frozen and stored at room temperature for 20 min before experimental trials. Mice were anesthetized with ether and the upper dorsal region was shaved with electric hair clippers and hair removal cream. Before *D. farinae* application, shaved dorsal skin and surfaces of each ear were washed with 150 μL of 4% sodium dodecyl sulfate (SDS) (Sigma) to remove the greasy component and destroy the cuticle barrier. After SDS application, the skin was dried by cold wind. Then, atopic dermatitis was induced by topical application (painting) of 100 mg *D. farinae* ointment on shaved dorsal skin and both surfaces of the back of ears. These procedures were repeated twice a week for three weeks. 

### 2.3. Preparation and Treatment of LGI3 

A LGI3 peptide corresponding to a pentamer was synthesized by solid phase synthesis using Peptron. LGI3 peptide concentration of 1 mg/mL was prepared by a solvent mixture of 99% PBS solution and 1% PEG 400. PEG 400 dissolute LGI3 in PBS prevents the loss of solution from the dorsal skin, and enhances uptake into skin tissues. The prepared LGI3 mixture was applied once to shaved dorsal skin once a time for one month after induction of atopic dermatitis with *D. farinae* ointment.

### 2.4. Evaluation of Atopic Dermatitis Skin Lesion

After 14 days of treatment, the dorsal skin and surfaces of ears were photographed by a digital camera (Samsung, Korea) with 1 mm^2^ graph paper, once every three days until the 15th day (0, 3, 6, 9, 12, 15). The severity of atopic dermatitis symptoms has been assessed by the scoring atopic dermatitis (SCORAD) method [25]. The day of AD skin recovery was recorded separately in each group. The degree of each symptom was as follows: (1) erythema/darkening; (2) edema/papulation; (3) oozing/crust; (4) excoriation; (5) lichenification; and (6) dryness (scored as 0 (none), 1 (mild), 2 (moderate), and 3 (severe). The overall dermatitis score was determined from the sum of all individual scores in a double-blinded manner. The number of pixels in darkening (discoloration) and lichenification area were traced by computer software, ‘Paint-NET’ and the difference in the number of pixels means variation in lesion size in each group. The property of AD skin lesions could be evaluated by % darkening (discoloration) and lichenification area obtained using the following formula:

% darkening (discoloration) and lichenification area = (Darkening (discoloration) and lichenification area/Total dorsal skin area) × 100.

The mice were sacrificed on day 15, and dorsal skin thickness was measured by Vernier calipers.

### 2.5. Histopathological Examination

Twenty-one NC/Nga mice were prepared for histopathological evaluations. All experimental mice were sacrificed on day 15. The mice were anesthetized with ether prior to removing the dorsal area. The excised dorsal skin from each group was washed with PBS, fixed in 10% paraformaldehyde solution for several days, and then dehydrated in 70% ethyl alcohol. After dehydration, the tissues were embedded in paraffin then cut into 5 μm slices. The sliced sections were stained with hematoxylin & eosin and Toluidine blue for light microscopy and image analysis (Leica, Wetzla, Germany).

### 2.6. Assessment of LGI3 Peptide Concentration

In the AD model group, the recovery of skin barrier is very poor, because the concentration of LGI3 peptide was low in blood. Serum was collected from the control and AD model group and analyzed for LGI3 peptide concentration using an ELISA Kit (CSB-EL012900MO; Cusabio, Wuhan, China). According to manufacturer’s directions, ELISA was performed. In brief, the antibody specific for LGI3 as pre-coated onto a microplate. Standards and serum samples were pipetted into the wells and LGI3 (if present) was bound by the immobilized antibody. After removing any unbound substances, a biotin-conjugated Horseradish Peroxidase (HRP) was added to the wells. Following a wash to remove any unbound avidin-enzyme reagent, HRP substrate 3,3′, 5,5′-Tetramethylbenzidine (TMB) was added to detect HRP enzymatic reactions, which was catalyzed from a blue to yellow product after adding a stop solution. The substrate was added to the wells and color developed in proportion to the amount of LGI3 bound in the initial step. The color development was stopped and the intensity of the color was measured. Results were expressed as pg/mL serum.

### 2.7. Immunohistochemical Evaluations

Immunohistochemistry was performed on the dorsal skin of experimental mice. Antibodies against mouse FLG was purchased from LifeSpan BioSciences (Seattle, WA, USA). Five μm sections were prepared from formalin fixed paraffin embedded tissue specimens, de-paraffinized, and rehydrated in graded alcohols. A heat induced epitope retrieval technique (by autoclaving slides for 3 min in 10 mM citric acid buffer) was used for the detection of FLG. After slides were quenched in endogenous peroxidase with 3% H_2_O_2_ in PBS for 10 min, they were incubated overnight with primary antibodies (Filaggrin, 1:200; Cell Signaling, Beverly, MA, USA). Afterward, solid phase absorbed rabbit Immunogen fraction (DakoCytomation, Denmark) was used to demonstrate the specificity of FLG. Visualization was performed using the LSAB+ kit (DakoCytomation) with 3,3′-diaminobenzidine tetrahydrochloride (DAB) as the chromogen according to the manufacturer’s instructions.

### 2.8. Statistical Analyses

The data and values are expressed as mean ± standard deviation (SD). ANOVA (analysis of variance) and Fischer’s exact test were used to determine the statistical significance of differences between groups. The statistical analyses were performed using SPSS Statistics 25 for Windows (IBM, Armonk, NY, USA). A *P* value below 0.05 indicates a significant difference. 

## 3. Results

### 3.1. LGI3 Concentration in Serum of Both Control and AD Group

**Figure 1:**
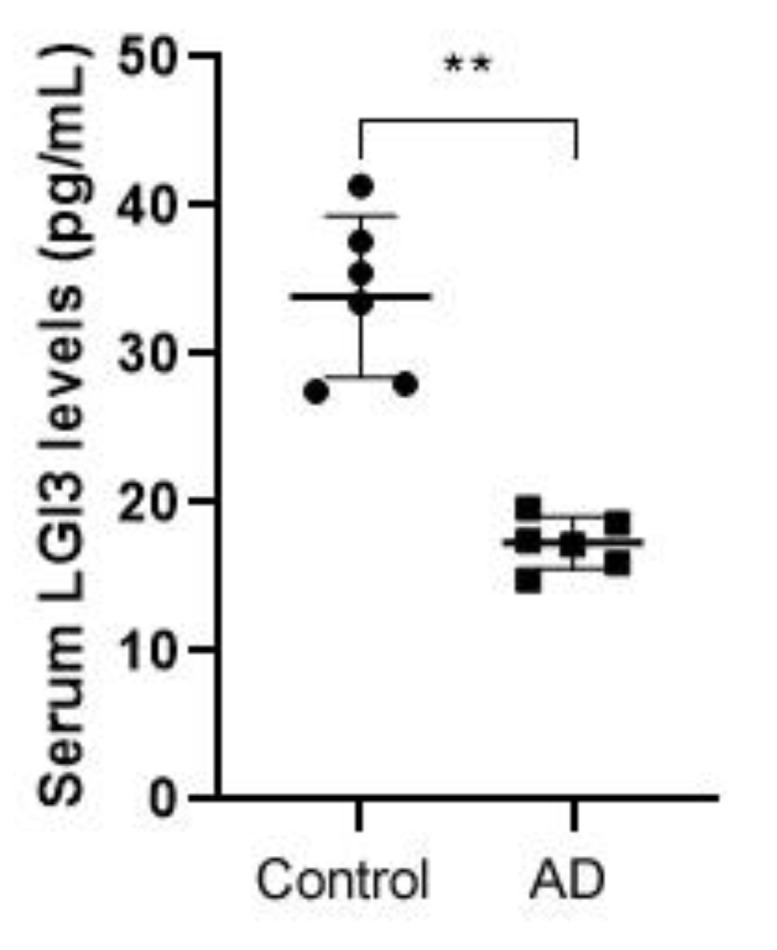
Comparison of leucine-rich glioma inactivated 3 (LGI3) concentration in serum of both the control and atopic dermatitis (AD) group (n = 6). The concentration was significantly reduced in the AD group compared with the control group. Data are expressed as mean ± S.D. (** *P* < 0.01 vs. control group). Differences between control and AD model group were estimated by ANOVA.

To identify the potential alleviating effects of LGI3 in AD, serum concentration was measured using an LGI3 ELISA. The concentration was significantly reduced in the AD group (16.65 ± 1.46 pg/mL, *P* < 0.01) compared with the control group (33.63 ± 6.92 pg/mL) (Figure 1). 

### 3.2. Effect of LGI3 Peptide Treatment on Atopic Dermatitis Skin Lesions 

**Figure 2:**
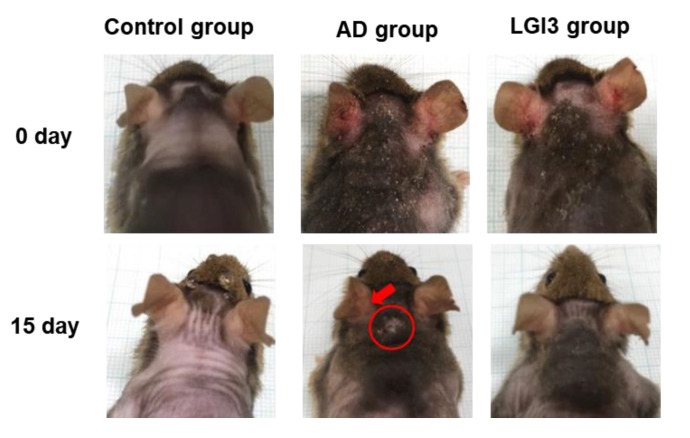
Representative photographs showing a comparison of atopic dermatitis (AD) skin lesions in the control, AD, and leucine-rich glioma inactivated 3 (LGI3) group (n = 7). The AD and LGI3 group are characterized by AD induction on day zero compared to the control group. LGI3 treatment attenuates AD compared to the AD group on day 15.

**Figure 3:**
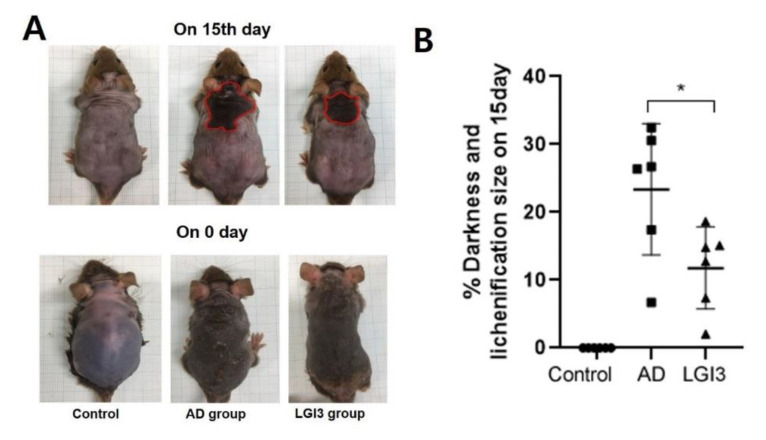
Effect of leucine-rich glioma inactivated 3 (LGI3) treatment on % darkness and lichenification area in mice (n = 6). (**A**) Chronic atopic dermatitis (AD) skin lesions area featured with darkness and lichenification. (**B**) Improvement in chronic AD skin lesions in LGI3 compared to AD group (**B**). The alleviating effects on darkening and the lichenification area in LGI3 group were significant compared to the AD group. Data are expressed as mean ± S.D. (* *P* < 0.05 vs. AD model). Differences between the control and AD model, LGI3 and the AD model group were estimated by ANOVA.

**Figure 4:**
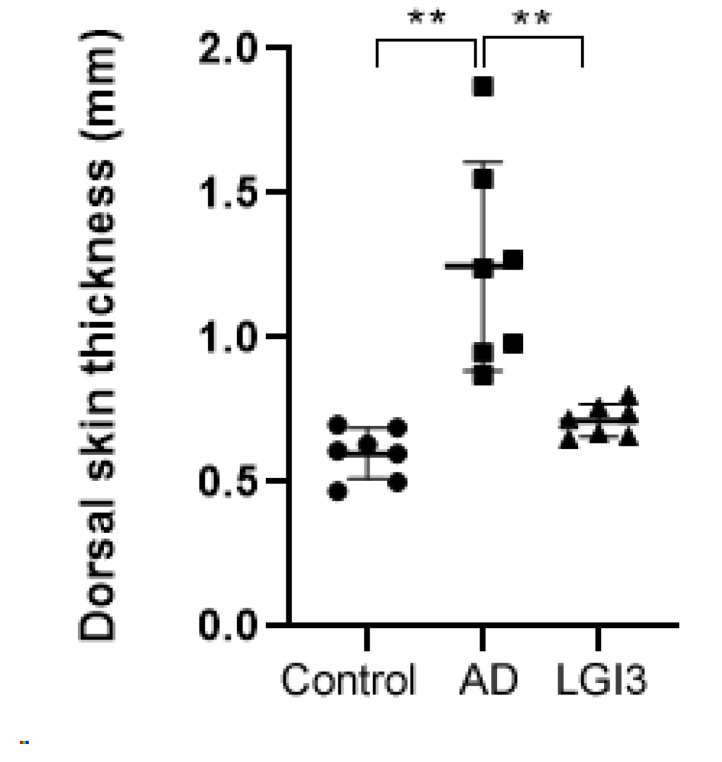
Dorsal skin thickness of mice from each group measured by a thickness gauge (n = 7). The dorsal skin thickness in the leucine-rich glioma inactivated 3 (LGI3) group was not as thick as the atopic dermatitis (AD) group (0.71 ± 0.06 mm vs. 1.25 ± 0.36 mm). Data are expressed as mean ± S.D. (** *P* < 0.01 vs. AD group). Differences between the control and AD model, LGI3 and AD model group were estimated by ANOVA.

Representative dorsal skin photographs of experimental mice are shown in Figure 2. AD symptoms induced by Biostir AD ointment were mitigated after topical application of LGI3 once a day for 15 days. AD skin lesions were obvious in AD and LGI3 groups at the first day of taking pictures prior to LGI3 treatment. Dry and keratinized skin has been noticed on the dorsal, in addition to severe erythema and excoriation on both surfaces of the back of ears. On day 15, the LGI3 group showed improvement on AD skin lesions compared to the AD group. For example, there were some variations in the alleviating effects such as erythema, darkening, excoriation, and keratinization on the back of ears. In the case of the dorsal skin, darkening and lichenification (representative clinical features of chronic AD skin lesions) were observed in both the AD and LGI3 group. However, the alleviating effects on the darkening and lichenification area in the LGI3 group (12.74 ± 7.37%, *P* < 0.05) were significant compared to the AD group (26.71 ± 6.72%) (Figure 3A,B). Furthermore, AD markedly promoted deterioration of dorsal skin including thickening as well as wrinkles. In the AD group, dorsal skin thickness was 1.25 ± 0.36 mm (*P* < 0.01). The result indicates that the AD group was significantly thicker than the control group (0.60 ± 0.09 mm). We found that the dorsal skin thickness in the LGI3 group was not as thick as the AD group (0.71 ± 0.06 mm, *P* < 0.01) (Figure 4). 

### 3.3. Effect of LGI3 Treatment on Atopic Dermatitis Clinical Symptoms in Mice

**Figure 5:**
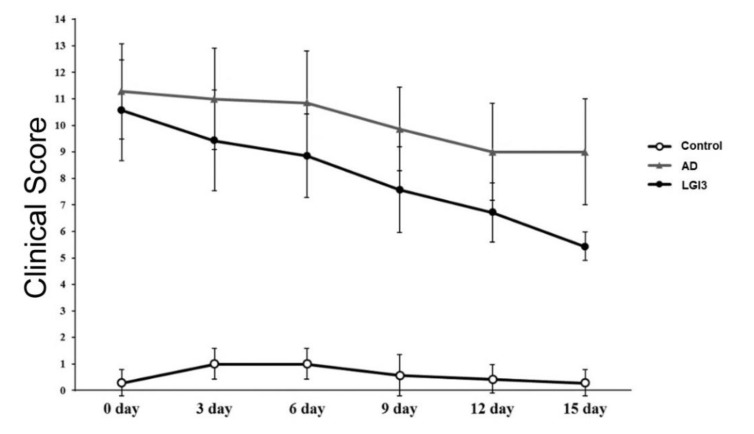
Clinical score of atopic dermatitis (AD) skin lesions from each group of mice (n = 7). On day 15, elevated scoring atopic dermatitis (SCORAD) Index was observed in the AD group (9 ± 2 score) compared to the control group. Treatment with the leucine-rich glioma inactivated 3 (LGI3) reversed this change (5.43 ± 0.53 score). Data are expressed as mean ± S.D. Statistical analysis was performed using ANOVA.

Severity of clinical symptoms of AD skin was measured by the SCORAD Index. On day 6, marked alleviation has been observed in the SCORAD index of the LGI3 group (mean ± SD = 8.86 ± 1.57 score) compared to the AD group (mean ± SD = 10.86 ± 1.95 score). On the other hand, the LGI3 group (5.43 ± 0.53 score) showed significant improvement in the SCORAD index in contrast to the AD group (9 ± 2 score) (ANOVA, *P* < 0.001) on day 15 (Figure 5).

However, some parts of dorsal skin in the LGI3 group developed chronic AD symptoms such as discoloration and lichenification, prior to treatment. Thence, the LGI3 group could not be assessed as the same clinical scores of the control group at the end of the experiment. 

### 3.4. Histopathological Examination of Epithelial Cell Layer of Dorsal Skin in Mice Treated with LGI3 Peptide

**Figure 6:**
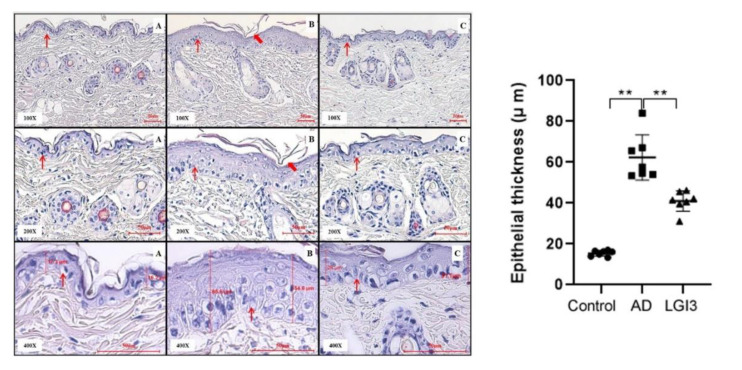
Thickness of epithelial cell layer of dorsal skin measured by the light microscopy. Representative photomicrographs of dorsal skin on day 15 from each group of mice: H&E staining (100×, 200×, 400×). (**A**) Dorsal skin of the control group; (**B**) dorsal skin of the atopic dermatitis (AD) group; (**C**) dorsal skin of the leucine-rich glioma inactivated 3 (LGI3) group. The thin arrow indicates the epithelial skin tissue and the thick arrow indicates parakeratosis. Remarkable suppressive effect has been demonstrated in abnormal epidermal thickness of dorsal skin (37.3 ± 8.75 μm, *P* < 0.01) in the LGI3 group (N = 7). Data are expressed as mean ± S.D. (** *P* < 0.01 vs. AD model). Differences between the control and AD model, LGI3 and AD model group were estimated by ANOVA.

Photomicrographs (100×, 200×, 400×) of dorsal skin from each group are displayed in Figure 6. H&E staining was conducted on day 15. The control group demonstrated well-aligned epidermal tissue. However, epidermal thickening and wrinkles were observed in the AD group due to hyperkeratosis and parakeratosis. Hyperkeratosis and parakeratosis revealed that the skin barrier function was abnormal. Although the LGI3 group was not transformed into normal skin (compared to control group), LGI3 treatment generates re-epithelialization and regeneration required for normal skin barrier function. Moreover, we confirmed the alleviating effect of abnormal skin barrier due to hyperkeratosis and parakeratosis compared with the AD group.

We measured the thickness of the epithelial cell layer of dorsal skin using the light microscopy analysis. The tissue thickness of the AD group (59.5 ± 11.14 μm, *P* < 0.01) was significantly higher than the control group (15.17 ± 2.11 μm) due to hyperkeratosis of the collapsed skin barrier. However, a remarkable suppressive effect was observed in abnormal epidermal thickness of the LGI3 group (37.3 ± 8.75 μm, *P* < 0.01) (Figure 6). These results demonstrate that LGI3 plays a critical role in repairing impaired skin barrier. 

### 3.5. Histopathological Examination of Mast Cell Infiltration into Dorsal Skin of Mice Treated with LGI3 Peptide

**Figure 7:**
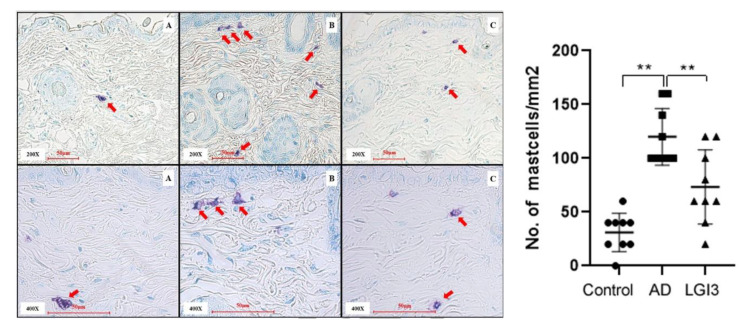
Leucine-rich glioma inactivated 3 (LGI3) treatment ameliorates granulated mast cells in atopic dermatitis (AD) models. Representative photomicrographs of dorsal skin from each group of mice on day 15 (Toluidine blue staining 200×, 400×). (**A**) Dorsal skin of control group; (**B**) dorsal skin of AD group; (**C**) dorsal skin of LGI3 group. Thick arrows indicate granulated mast cells infiltrated into the skin tissue. The number of granulated mast cells was within 1 mm^2^. Infiltration of granulated mast cells in the AD model (11.00 ± 3.46, *P* < 0.01) was conspicuously much more than the control group (2.29 ± 0.95) and the LGI3 group (4.57 ± 2.15) (N = 7). Data are expressed as mean ± S.D. (** *P* < 0.01 vs. AD group). Differences between the control and AD model, LGI3 and AD model group were estimated by ANOVA.

Photomicrographs (200×, 400×) of dorsal skin from each group of mice are presented in Figure 7. Toluidine blue staining was conducted on day 15. We found a number of mast cell infiltrations in the AD group compared with the control and LGI3 group. The total number of granulated mast cells within 1 mm^2^ was counted. Infiltration of granulated mast cells in the AD model (11.00 ± 3.46, *P* < 0.01) was conspicuously much more than the control (2.29 ± 0.95) and the LGI3 group (4.57 ± 2.15) (Figure 7). These results indicate that LGI3 plays a central role in suppressing mast cell infiltration.

### 3.6. Immunohistochemical Evaluation of Filaggrin Expression in Epithelial Layer of Dorsal Skin of Mice Treated with LGI3 Peptide

**Figure 8:**
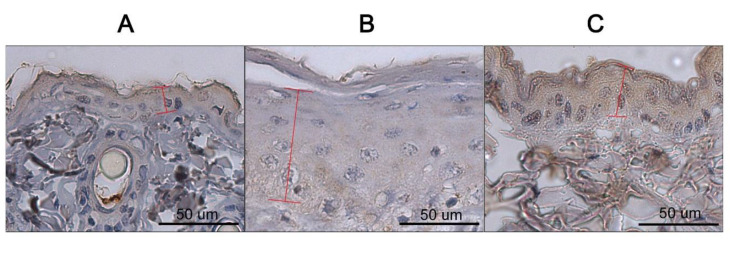
Representative photomicrographs of dorsal skin from each group of mice on day 15 (Filaggrin antibody staining 400×). (**A**) dorsal skin of the control group; (**B**) dorsal skin of the atopic dermatitis (AD) group; (**C**) dorsal skin of the leucine-rich glioma inactivated 3 (LGI3) group. Brown stained part in the skin epithelium is from filament aggregating protein (FLG). A red indicator: Epithelial skin tissue. In the control group, FLG was evenly expressed in the epidermis, and the LGI3 group showed a high intensity of brown staining color in the epidermis.

Photomicrographs (400×) of the dorsal skin from each group of mice are shown in Figure 8. We conducted immunohistochemical staining for FLG on the epithelial cell layer of dorsal skin on day 15. FLG was visualized with 3,3′–diaminobenzidine tetrahydrochloride (DAB, brown). FLG (essential for regulation of epidermal homeostasis) is a marker of skin barrier function and AD skin. In the control group, FLG was evenly expressed in the epidermis, and the LGI3 group showed a high intensity brown staining color in the epidermis. However, the AD group showed a noticeable difference from other groups. In the AD group, the epidermis was thicker than others, and showed low intensity staining. 

## 4. Discussion

In this study, we demonstrated that the LGI3 concentration in serum was downregulated in the AD murine model. This result suggests that enhanced skin barrier function by LGI3 could be a key component for the pathophysiology of AD. When the skin barrier is impaired, water loss is accelerated and the lesion is prone to *Staphylococcus aureus* invasion [26,27]. Therefore, restoring the skin barrier is essential in AD treatment. Topical application of LGI3 onto the skin surface has attenuated the clinical score of AD symptoms. In particular, improvement in dryness has been evident with the most significant difference between the LGI3 and AD group. Furthermore, topical LGI3 was able to improve the chronic symptoms of AD including darkness, lichenification, and dorsal skin thickness. Chronic symptoms of AD might turn into irreversible scars if the symptoms progress to severe without proper treatment. The findings in the present investigation suggest that LGI3 might protect the skin from chronic AD symptoms. 

Normal skin expresses differentiation markers that maintain skin barrier homeostasis. However, dysfunction of skin barrier is a major feature of AD patients, whose differentiation markers are downregulated. AD patients not only show acute lesions (such as erythematous papules, blisters, epidermolysis, and severe exudative lesions), but also chronic lesions such as darkening, wrinkle, and lichenification. Additionally, the skin may become drier than normal due to water loss from impaired skin barrier. 

AD skin lesions are associated with one or more typical atopic signs. For instance, not only are hyperkeratosis and parakeratosis shown in acute lesions, but also marked epidermal hyperplasia, acanthosis, and accumulation of mast cells such as in chronic lesions [1]. In AD skin barrier, excessive epidermal growth (due to incomplete re-epithelialization and regeneration) leads to these lesions. We demonstrated that the AD group showed epidermal hyperplasia including hyperkeratosis and parakeratosis compared with the LGI3 group that ameliorated the epidermal lesions. Furthermore, we found that LGI3 could suppress the invasion of mast cells into AD skin. The mechanism of suppression may not be related to the direct inhibition of immune response, but could be attributed to the recovery of skin barrier function. TSLP accelerates its expression when the skin barrier collapses and FLG decreases. Conversely, reduced expression of TSLP through restoring the barrier function of the skin could switch off the immune response of AD (24401911). Therefore, further studies are needed to examine the effects of LGI3 on TSLP expression in keratinocytes.

LGI3, a cytokine produced by keratinocytes, plays an essential role in promoting differentiation. Previous studies have demonstrated that LGI3 increases the expression of skin barrier differentiation markers including FLG. However, the mechanism of expression of differentiation markers by LGI3 has not been elucidated yet. The mechanism of LGI3 might be related to calcium dependent keratinocyte differentiation, as calcium is the key element, which strongly induces keratinocytes that form the outer most layer, the barrier function of the skin. 

The AD healing process includes re-epithelization and differentiation of keratinocytes in terms of skin barrier function recovery. Skin re-epithelization is an important component of wound healing including AD skin lesions. Incomplete and excessive re-epithelization results in additional skin lesions such as hyperkeratosis and parakeratosis. The key factor of these problems is proliferation and differentiation of keratinocytes. FLG is an important regulator of epidermal homeostasis. Loss-of-function mutations within the FLG gene have greatly increased the risk of AD and are considered as the most frequent genetic defects causing AD. For that reason, FLG has the potential to improve the quality of care for AD patients and ultimately preventing the development of the disease.

Although the mechanism of increment in FLG by LGI3 has still not been definitively elucidated, and the attenuating effect on AD might be due to the increment in FLG by LGI3, which affects the restoration of the skin barrier. In a previous study, treatment with LGI3 induced the expression of differentiation markers (including FLG) in HaCaT cells in a concentration-dependent manner [22]. Additionally, the LGI3 group showed a high intensity of the FLG antibody staining. In contrast, the AD group (with damaged skin barrier function) had a reduced intensity of FLG. Herein, immunohistochemistry revealed that impaired expression of FLG was observed in thickening of the AD skin barrier. However, treatment of LGI3 reversed this change. This result suggests that LGI3 upregulates FLG expression, resulting in attenuation of the thickening of the AD skin barrier. However, further studies are required to examine the role of LGI3-regulated FLG using in vivo transfection with FLG siRNA or FLG knockout mice.

## 5. Conclusions

The AD mouse model displayed a low level of LGI3 peptide in serum and damaged skin barrier. Thus, this study hypothesized that suppression of LGI3 appears to be closely associated with impairment of skin barrier in AD, and treatment with LGI3 might be crucial to restore the damaged skin barrier in AD.

The outcomes assessed from photographs, clinical score, and dorsal skin thickness indicate that treatment with LGI3 could heal skin faster than other groups receiving no medication. On the other hand, histopathological examination displayed that LGI3 is involved in re-epithelialization and regeneration of skin barrier through keratinocyte differentiation compared to the AD model (characterized by abnormal and incomplete skin barrier). From immunohistochemical evaluation, LGI3 can protect the AD skin barrier via increasing the expression of FLG.

In conclusion, LGI3 results in a suppressive effect on skin barrier dysfunction in the AD murine model. The ameliorating effect of LGI3 might help to restore skin barrier function and maintain skin barrier homeostasis. Hence, LGI3 might be considered as a new potential therapeutic target to restore skin barrier function in AD.
